# Uncovering knowledge on travel behaviour during COVID-19: a convergent parallel mixed-methods study in the context of Italy

**DOI:** 10.1007/s43039-021-00036-7

**Published:** 2021-08-18

**Authors:** Giacomo Del Chiappa, Ilenia Bregoli, Marcello Atzeni

**Affiliations:** 1 Department of Economics and Business, University of Sassari, Via Muroni, 25, 07100 Sassari, Italy; 2 Lincoln International Business School, University of Lincoln, Lincoln, UK; 3 Department of History, Human Sciences and Education, University of Sassari, Via Zanfarino, 62, 07100 Sassari, Italy

**Keywords:** Protection motivation theory, Travel behaviour, Coping strategies, COVID-19, Mixed method, Convergent parallel design, Italy

## Abstract

Against the background of uncertainty and crisis generated by COVID-19, academics and practitioners have struggled to envision how travelling behaviour will be transformed by the pandemic and when it will resume. Despite its relevance to both theory and practice, current research devoted to this research strand is still in its early stages. This study, reliant on Protection Motivation Theory, was conducted in order to assess the ways in which travellers’ preferences are changing as a result of the coping strategies they adopt to protect themselves from the health crisis. To do this, a convergent parallel mixed method approach (data validation variant) was applied to a sample of 4,539 completed questionnaires, collected in Italy, which included 1,577 usable qualitative answers. A factor-cluster analysis was carried out on the quantitative data. Two factors driving destination choice emerged, namely: “Personal protective equipment, sanitation, and physical distancing” and “Outdoor and under-crowded tourism attractions and destinations”. The cluster analysis divided individuals into three groups: “All-round concerned tourists”, “Middle-concerned tourists”, and “Outdoor-driven tourists”. Finally, a series of chi-square and F-tests revealed that significant differences existed between the clusters, based on socio-demographics and travel-related characteristics (i.e., preferred accommodation facilities and means of transport, geographical scale of travelling, and travel companions). Quantitative results were then merged with qualitative results, allowing us to further deepen our understanding of travel behaviours during the pandemic and the related coping strategies. Contributions to this body of knowledge and managerial implications are discussed and suggestions for further research are given.

## Introduction

The COVID-19 global outbreak has forced many tourism destinations to cease operation, either partially or totally, due to lockdown measures, travel bans, and the lack of confidence of tourists who have postponed their holidays in recent months (Sigala, 2020). In these extremely uncertain times, enhancing our understanding of how travel behaviour has changed because of the pandemic is vital for both the academia and the industry. So far, however, only a handful of articles have addressed this issue (Zenker & Kock, 2020). For example, recent research has shown that individuals are more willing to travel domestically, favouring proximity tourism to easily accessible destinations (e.g., Lew et al., 2020), with many practicing mountain tourism, second-home tourism, and outdoor tourism (Seraphin & Dosquet, 2020). Furthermore, it has been suggested that tourists are likely to seek out destinations in which well-established infrastructures and high-quality medical facilities are available (e.g., Wen, Kozak, Yang, and Liu, 2020). Research also suggests that individuals mostly travel independently or in small groups (Wen, et al., 2020).

In the light of the above, it is necessary to empirically expand our knowledge on (new) tourists’ behaviour. This is useful, not only in advancing the current body of knowledge on tourists’ behaviour during the time of COVID-19, but also in providing practitioners with useful information with which they can plan changes to their operations so that newly emerging customers’ needs and wants can be satisfied (Del Chiappa, Pung, and Atzeni 2021; Gursoy et al., 2021; Gursoy & Chi, 2020; Sigala, 2020; Zenker & Kock, 2020). This, in turn, could prove to be a relevant and useful way of supporting the restarting, reformation, and reimagining of the tourism and hospitality industry, assisting in the recovery of the whole economy, which has experienced a global recession as a result of the “butterfly effect” that COVID-19 has generated (Lacroix & Milliot, 2020). So far, to the best of the authors’ knowledge, no prior studies have sought to understand how consumers’ preferences in terms of destination selection criteria are changing as a consequence of self-protective attitudes and behaviours generated by the COVID-19 pandemic. These attitudes are expected to last until a vaccine renders the crisis over. In an attempt to address this knowledge gap, this paper aims to profile a sample of Italian travellers, based on their destination selection criteria, and ascertain whether significant differences exist among them based on their socio-demographics (i.e., age, gender, level of education, and employment status) and travel-related variables (preferred accommodation facilities and means of transport, geographical scale of travelling, and travel companions). In addition to this, the paper also aims to investigate how travellers differ with regards to the coping strategies that they have adopted towards travel during the pandemic. In order to do this, Protection Motivation Theory (hereafter PMT) was adopted (Wang et al., 2019; Zheng, Luo, and Ritchie, 2021). Unlike previous studies that have applied PMT to the study of tourists’ behaviour during this pandemic, our study focuses solely on the analysis of the different coping behaviours adopted by travellers, facilitating a better understanding of whether these coping behaviours vary based on tourists’ socio-demographic attributes or travel-related variables. Given the exceptional nature of the COVID-19 pandemic, this study was informed by the desire to enhance our understanding of different coping strategies, seeking to provide a more granular knowledge of aspects that even recent research has failed to address, given the fact that the items measuring coping were too broad (e.g., Zheng, Luo, and Ritchie, 2021a). Indeed, as He and Harris (2020) have pointed out, it is important to better understand consumers who may be more willing to pursue pleasant experiences following the pandemic, not just within tourism, but in relation to their broader consumption behaviours. Furthermore, the same authors highlighted the uncertainty surrounding the behaviour of tourists and the need to study the impact that the pandemic has had on their choices, such as whether or not they plan to travel again soon, whether they might gradually start travelling again due to fear, or whether they plan to minimise travel or re-evaluate their travel choices by favouring sustainable travel practices (He & Harris, 2020).

For the purposes of the study, a convergent parallel mixed method, based on a data validation variant, was applied. Specifically, data was collected through a survey that included both closed and open-ended questions, with results from the open-ended questions used to confirm/validate/deepen the results of the closed-ended questions (Creswell and Plano Clark, 2017). A convenience sample of 4,539 completed questionnaires were collected (using a snowball sampling technique), including 1,577 usable qualitative answers. The two strands of research were therefore examined separately (i.e., quantitative data: factor-cluster analysis; qualitative data: thematic analysis) and then merged to relate/validate results. According to existing literature (e.g., Pechmann et al., 2003) PMT-related studies have hitherto primarily adopted either quantitative approaches (e.g., surveys and experiments) or qualitive ones. The desire to overcome the limits of each approach when separately conducted (i.e., offering a partial view), coupled with the need to gather as many details as possible about latent and emerging travelling behaviours related to the unknown and extremely dynamic environment shaped by COVID-19, justifies the choice to adopt a convergent parallel mixed method. This also represents one of the main contributions of the study, given that, to the best of the authors’ knowledge, mixed methods have not been applied in PMT-related studies so far.

This paper is structured as follows: a literature review will briefly present the main constructs of the PMT, along with the results of recent research on tourists’ behaviour during COVID-19 pandemic. The methodology section then presents the convergent parallel mixed method design developed for this research and, following this, the results of the quantitative and qualitative strands of research are presented. A discussion, in which both the quantitative and qualitative strands of research are merged, is then offered. Conclusions, theoretical and managerial implications, limitations, and future research avenues are then presented.

## Literature

### Protection motivation theory

PMT was developed in the context of health in order to enable an understanding of the influence of fear on attitudinal and behavioural changes, particularly those related to health-related issues and diseases (Floyd et al., 2000). This theory has been applied to the study of tourists’ behaviours during times of health crisis (Wang et al., 2019) and, more recently, to the study of tourists’ behaviours during the current COVID-19 pandemic (Rather, 2021; Zheng, Luo, and Ritchie, 2021a).

The Protection Motivation construct represents the intention of a person to perform a suggested behaviour, thus constituting a behavioural intention that develops as a result from coping and threat appraisal processes (Norman et al., 2005). Coping appraisal refers to the likelihood of an individual adopting adaptive behaviours (e.g., following given advice) that depend upon the belief that the adaptive behaviours will be effective (i.e., response efficacy), that the individual is capable of adopting them (i.e., self-efficacy) and the perceived costs associated with adopting these adaptive behaviours (Pechmann et al., 2003). Threat appraisal, on the other hand, refers to the probability of an individual adopting a maladaptive behaviour, i.e., an individual, instead of dealing with a threat, might prefer to take action to reduce the fear associated with it; for instance, as in the case of an individual who denies a threat (Norman et al., 2005). Maladaptive behaviours are said to develop when the perceived vulnerability of an individual is high, the efficacy of responses is low, and there are benefits associated with the maladaptive behaviour (Floyd et al., 2000; Norman et al., 2005). The results of these processes can differ and these outcomes can be categorized into six coping methods when it comes to danger (Rippetoe & Rogers, 1987). The first is associated with adaptive behaviours, while the other five are related to maladaptive behaviours: (1) rational problem solving, in which an individual analyses a problem in order to come up with a solution, e.g., shifting their preferences and adopting a different way of travelling; (2) religious faith and religious beliefs used to cope with danger, e.g., continuing to travel and entrusting one's life to God’s protection (Ben-ahron et al., 1995); (3) avoidance, e.g., continuing to travel as before the pandemic by denying any possible danger that might affect holiday spirit (the relaxation, enjoyment, and fun experienced by travellers on holiday, as defined by Wang et al., 2019); (4) wishful thinking, e.g., hoping that the availability of the vaccine and its distribution to the wider population will make travelling possible again, making personal travel behaviour changes unnecessary (Ben-ahron et al., 1995); (5) fatalism, e.g., continuing to travel while accepting the situation shaped by the pandemic and entrusting one’s life to fate as there is nothing that can be done to cope with COVID-19; and (6) hopelessness, e.g., the feeling it is almost useless to try to do something to prevent health risks when travelling (Rippetoe & Rogers, 1987).

In this paper, rather than applying the full PMT, we have decided to limit our focus to adaptive and maladaptive behaviours in an attempt to understand them in more depth. In doing so, our research differentiates itself from previous studies that have applied PMT to the study of tourists’ behaviour.

### Tourist behaviour during the COVID-19 pandemic

Research on the impact of the pandemic on tourists’ behaviour has been increasing in an attempt to understand how behaviours are affected by this unprecedented crisis. In general, studies have found that tourists would still travel if they felt that they had control over the different elements of their travelling (Sánchez-Cañizares et al., 2020) and if they displayed a motivation to protect themselves (Zheng et al., 2021a). Research has mainly focused on three areas: (1) identification of favoured destinations, (2) travel party, and (3) chosen services.

Recent findings from research conducted on a US sample of respondents showed that the pandemic has led to the avoidance of foreign travel from both a short- and long-term perspective (Chua et al., 2020). Similar findings were reported in a study on a sample of Chinese respondents, who preferred to travel only a short distance to their destinations (Zheng et al., 2021a). Other pieces of research focusing on understanding tourists’ choices with regards to destinations found that tourists preferred to travel domestically, favouring proximity tourism to destinations less affected by COVID-19, and to places that were less crowded (Aiello, Bonanno, and Foglia, 2020), easily accessible (e.g., Lew et al., 2020), already known (Kock et al., 2020), and where well-established infrastructures and high-quality medical facilities were available (e.g., Wen et al., 2020). Furthermore, recent studies reported tourists choosing destinations allowing them to practice mountain tourism, second-home tourism, and outdoor tourism (Seraphin & Dosquet, 2020).

As far as tourists’ preferences in terms of their travel party are concerned, existing studies report some contradictory findings. Indeed, some research has reported that tourists prefer to travel independently or in small groups (Wen et al., 2020; Zheng et al., 2021a), while other studies found that participants would prefer to travel in a group, preferably in an organised travel (Kock et al., 2020).

With regards to the types of services chosen by tourists, it was found that the likelihood of a tourist choosing a hotel or B&B depended on the importance that he/she placed on security (the higher the importance, the more likely it was that the tourist would choose to stay in a hotel or a B&B) and the place of residence (tourists from highly affected areas would be less likely to go to hotels or B&Bs) (Aiello et al., 2020). Existing literature also showed that, during this pandemic, individuals have tended to use more private cars when compared to other means of transport (e.g., planes: Perić, Dramićanin and Conić, 2020), especially when travelling for relatively short distances (e.g., Abdullah et al., 2020). Other studies have claimed that tourists prefer to purchase travel insurance (Kock et al., 2020) and pay more attention to the cleaning and social distancing practices adopted. For instance, on the last point, research utilising experiments highlighted that tourists’ perceived health risks to be lessened if their hotel offered advanced cleaning procedures and used technologies facilitating social distancing, such as digital check-in procedures (Shin & Kang, 2020). Moreover, Addo et al. (2020) reported customers’ interest in using personal protective equipment (PPE: e.g., face masks, hand sanitiser, etc.) to cope with the health risks caused by COVID-19.

In addition to the aforementioned factors, recent research has also investigated the link between some socio-demographic variables and tourists’ risk perceptions, as well as their future travel intentions. For instance, older tourists were shown to have a higher risk perception and less of an intention to travel than younger tourists (Peluso & Pichierri, 2020). Similarly, women also appeared to have a higher perception of risk (Neuburger & Egger, 2020). In addition to this, it was also found that tourists’ perceptions of risk are not static, but rather they change over time. For instance, Neuburger and Egger (2020) found that the clusters that they identified based on perceptions of the pandemic, perceptions of travel risks, and travel behaviour changed in the two phases of their research and, in the second stage, a cluster of “anxious” tourists emerged which did not exist in the first stage, which was conducted at the beginning of the pandemic.

However, it is useful to point out that the aforementioned literature reviewed studied the impact of the pandemic on tourists’ behaviour by applying quantitative methods (mainly surveys and, in very few cases, experiments). Hence, our study, in adopting a convergent parallel mixed method, allows us to gain a more in-depth understanding of respondents’ travel preferences during the pandemic, and, in this way, shed additional light onto this topic in a way that would not be possible if only one type of data had been collected.

## Methodology

For the purposes of this study, a convergent parallel mixed method, based on a data validation variant, was applied. In other words, researchers collected data through a survey made up of both closed- and open-ended questions, with results from the open-ended questions being used to confirm/validate/deepen the results of the closed-ended questions (Creswell and Plano Clark, 2017). This was carried out in light of the extremely dynamic environment created by the pandemic in order to gather updated and more thorough information regarding the “latest” trends in the travelling preferences of Italians.

The survey was comprised of four sections. The first part was developed based on existing literature (Lew et al., 2020; Seraphin & Dosquet, 2020; Wen et al., 2020) and sought to rate, on a 5-point Likert scale, the level of importance given to 9 destination selection criteria considered when choosing a holiday destination during the pandemic (1 = not at all important, 5 = extremely important). Specifically, this pertained to items related to personal protective equipment, sanitation measures, and physical distancing rules adopted at both the destination and at tourism businesses (6 items); the type of tourism destination (un-crowded, rural areas, etc.); and the amount of outdoor activities and open air-attractions at the destination (3 items). The second section included a list of 11 statements specifically chosen to investigate the geographical scale of travel (i.e., regional, national or international), the travel companion (i.e., solo travel, organized group, small group of friends, family), and the type of transport to be used (i.e., owned car, train/bus, plane, ferries/cruise ships). These items were measured using a 5-point Likert scale (1 = extremely unlikely, 5 = extremely likely). The third section included two open-ended questions: one asked the respondent to indicate their preferred destinations for future holidays and the reason for their choice, and the second asked them to explain how their travel would change as a consequence of the pandemic. The final section asked respondents to provide some general socio-demographics (i.e., age, gender, level of education, employment status) and travel-related characteristics (i.e., their preferred type of accommodation and the length of their holiday).

The survey was administered online through a snowball sampling technique. According to existing scholarly literature, this sampling technique is often used when subjects are difficult to locate and approach (Auerbach and Silverstein, 2003). Although the snowball sampling technique is not considered a random sampling approach, it was considered to be the best sampling approach for this research as it allowed us to collect data from a large sample of individuals across different regions in Italy (including those from remote areas). It also enabled us to cope with the financial constraints of this project (Wrenn, Stevens and Loudon, 2007) and the social distancing rules and travel restrictions imposed by the Italian government. The initial sample was generated from 2,000 contacts provided by an Italian tourism association. The data collection was supported by several tourism businesses (accommodation facilities, travel agencies, tour operator, airports, etc.), who sent out an email invitation to their newsletter subscribers and followers and promoted the survey on their social media profiles. Potential respondents received an email inviting them to complete an online survey by clicking on a link. They were encouraged to forward the survey to their friends and acquaintances. At the end of the data collection period (10th to 31st May), 4,539 complete questionnaires were obtained, including 1,577 narratives to be used for thematic analysis.

Quantitative data was analyzed using a factor-cluster approach and conducting a series of F-test and Chi-squared tests (SPSS Ver. 21). Qualitative data was read in order to familiarise researchers with the data, aiding thematic analysis (with the support of NVivo 12). Codes were developed from this data and, once identified and classified, were reviewed by the research team and subsequently by an independent researcher not involved in the study, whose role was to revise the coding and decide whether he/she agreed with the codes. Whenever a disagreement arose regarding a certain code, discussions were held until an agreement was reached and the final coding was approved. In addition to this, in order to merge the two strands of research, results from the cluster analysis were entered into NVivo12. In this way, it was possible to carry out a further analysis through a matrix query, allowing the themes identified in the qualitative analysis to be linked with the clusters identified in the quantitative analysis (i.e., “All-round concerned tourists”, “Middle-concerned tourists”, and “Outdoor-driven tourists”).

## Findings

### Quantitative findings

Table 1 shows the socio-demographic profile of respondents. Respondents were mostly female (64.4%), aged 25–54 years (25–34 = 21.6%; 35–44 = 27.2%; 45–54 = 24.2%), with a Masters’ Degree or PhD (41.5%), self-employed (46.9%), or retired (23.0%). They were reported to preferer 4–6 nights stays (39.8%) in 5-star/5-star superior hotels (18.5%) or camping (22.5%).

**Table 1:** Overall profile of the sample (%)

**Gender**		Unemployed	4.7
Male	35.6	Student	5.9
Female	64.4	Other	12.2
**Age**		**Type of accommodation you would choose in the next 12 months (2020–2021)**	
18–24	10.5	1-star hotel	8.8
25–34	21.6	2-star hotel	0.3
35–44	27.2	3-star/3-star superior hotel	0.4
45–54	24.2	4-star/4-star superior hotel	13.3
55–64	12.1	5-star/5-star superior hotel	18.5
over 64	4.4	Bed & Breakfast	2.9
**Level of education**		Camping	22.5
Secondary school	1.2	Holiday village	5.6
High school	3.6	Holiday home	3.1
University Degree	38.6	Other	24.6
Masters’ Degree/PhD	41.5	**Length of stay of your holidays in the next 12 months (2020/2021)**	
Other	15.1	1–3 nights	19.2
**Employment status**		4–6 nights	39.8
Employed	7.3	7–10 nights	25.0
Self-employed	46.9	11 nights or more	16.0
Retired	23.0		

A factor-cluster analysis (principal component analysis and varimax rotation) was carried out on the nine items devoted to measuring the selection destination criteria. Two factors driving destination choice emerged (42.52% of total variance).

The KMO index (Kaiser–Myer–Olkin = 0.824) and the Bartlett's test of sphericity (chi-square = 11,612.211; *p* value < 0.0001) were used to confirm that the results were appropriate in explaining the data. Cronbach's alpha was then calculated to test the reliability of the extracted factors; all values were 0.6 or higher, suggesting that the factors were reliable (Nunnally and Bernstein, 1994). Furthermore, in accordance with Hair, Black, Babin, and Anderson (2014), we checked for cross loadings higher than 0.4 and none emerged (see Appendix 1). For this reason, we decided to retain all of the items included in the factor analysis. “PPE, sanitation, and physical distancing” (29.03% of total variance) includes items describing measures and services that destinations and related tourism businesses could/should implement to guarantee safety, hygiene, and physical distancing and avoid excessive overcrowding in tourist areas. “Outdoor and under-crowed tourism attractions & destinations” (13.49% of total variance) is related to items describing the tourists’ preference to experience holidays in less crowded destinations and practice outdoor activities (Table 2).

**Table 2:** Results of factor analysis

	Mean	Loadings	Eigenvalue	% Var	% Var Cum	Alpha
*Factor 1: PPE, sanitation, and physical distancing*			2.613	29.03	29.03	0.812
The tourism destination has good healthcare infrastructures that can be easily accessed when needed	4.297	.602				
Public areas are properly cleaned and sanitised	4.410	.692				
Local authorities know how to manage social gatherings and guarantee social distancing standards in public and tourist areas	4.178	.813				
The access to public areas (e.g., beaches) can be booked (through an app, etc.) to properly manage social gatherings and prevent overcrowding in these places	3.740	.623				
Tourism businesses working within the area allow their customers to use contactless payment methods	3.570	.437				
Tourism businesses working within the area provide their customers with face masks and hand sanitiser	3.940	.662				
*Factor 2: Outdoor and under-crowed tourism attractions & destinations*			1.214	13.49	42.52	0.614
The tourism destination is not a crowded, well-known, and well-established tourist area (e.g., rural areas)	4.019	.542				
The tourism destination provides visitors with the option to enjoy several outdoor activities and experiences	4.408	.653				
The tourism destination has several open-air attractions	4.082	.545				

KMO = 0.824—Bartlett test: Chi-squared = 11,612.211; p-value = 0.000

The scores of the two principal components were entered into a cluster analysis. A double-step cluster method was used. A hierarchical cluster (Ward method—Manhattan distances) (Hair et al., 2014) was performed first. The dendrogram inspection allowed us to identify that the biggest increase in the distance between clusters existed between clusters 2 and 3, thus highlighting that the three cluster-based solution was able to create homogeneous groups. An ANOVA test (*p* value < 0.000) confirmed this finding.

A non-hierarchical cluster analysis was then performed using only the factor scores (k-means method). We tested the 2, 3, and 4 cluster solutions and we examined the group associations, group sizes, and dendrograms. The findings highlighted that the three cluster solution created more homogeneous groups in comparison to other solutions. Each of the three clusters showed distinct differences in their selection criteria items, and the ANOVA test (*p* value < 0.000) confirmed the validity of three cluster-based solution (Table 3) (Hair et al., 2014).

**Table 3:** Results of cluster analysis

	All-round concerned tourists	Middle-concerned tourists	Outdoor-driven tourists	F	Sig
	N = 2507	N = 1120	N = 912		
*Factor 1: PPE, sanitation, and physical distancing*	.480	.037	-136.766	3,572.072	.000
The tourism destination has good healthcare infrastructures that can be easily accessed when needed	4.63	4.33	3.35	759.223	.000
Public areas are properly cleaned and sanitised	4.77	4.43	3.41	1,198.735	.000
Local authorities know how to manage social gatherings and guarantee social distancing standards in public and tourist areas	4.70	4.17	2.75	2,358.734	.000
The access to public areas (e.g., beaches) can be booked (through an app, etc.) to properly manage social gatherings and prevent overcrowding in these places	4.27	3.56	2.50	983.920	.000
Tourism businesses working within the area allow their customers to use contactless payment methods	4.05	3.10	2.82	520.796	.000
Tourism businesses working within the area provide their customers with face masks and hand sanitiser	4.50	3.63	2.79	1,188.070	.000
*Factor 2: Outdoor and under-crowed tourism attractions and destinations*	.390	-100.542	.162	2,745.032	.000
The tourism destination is not a crowded, well-known, and well-established tourist area (e.g., rural areas)	4.50	3.13	3.77	857.804	.000
The tourism destination provides visitors with the option to enjoy several outdoor activities and experiences	4.77	3.44	4.60	1,522.988	.000
The tourism destination has several open-air attractions	4.43	3.19	4.22	757.756	.000

The final clusters were named: “All-round concerned tourists” (N = 2,507), “Middle-concerned tourists” (N = 1120), and “Outdoor-driven tourists” (N = 912). Table 3 shows the three cluster-based solution and provides the mean value for each item used to run the factor analysis. This was explicitly done to further support and improve the interpretation of each cluster.

Furthermore, a multiple discriminant analysis using a bootstrapping method confirmed the validity of the three-based cluster solution, with 96.9% of cases correctly classified (bootstrap procedure: 97.4% hits) (Table 4).

**Table 4:** Results of multiple discriminant analysis

Clusters	Group centroids	
	Function1	Function2
COVID-19 concerned tourists	− 0.516	1.034
Middle concerned tourists	3.601	0.880
Outdoor-driven tourists	1.015	1.554
Eigenvalue	4.334	2.090
Canonical correlation	0.880	0.801
Wilk’s Lambda	0.070	0.455
Chi-square	554.880	324.532
Significance	.000	.000

Classification results				
Actual group	Number of cases	Predicted group membership		
		COVID-19 concerned tourists	Middle concerned tourists	Outdoor-driven tourists
All-round concerned tourists	2,507	2,423 (96.6%)	64 (2.6%)	20 (0.8%)
Middle concerned tourists	1,120	33 (2.9%)	1,078 (96.2%)	9 (0.9%)
Outdoor-driven tourists	912	0 (0.0%)	15 (1.7%)	897 (98.3%)

Interpretations of each cluster were made by analysing the factor scores and the mean values related to each of them. Furthermore, in order to better understand the characteristics of each cluster, the mean value of the items was analysed for each (Table 3).

“All-round concerned tourists” (N = 2,507) was the largest cluster. People belonging to this group scored high or very high in all of the items (M > 4.0). In particular, they were mainly concerned with the cleaning and sanitisation of public areas (M = 4.77) and with the ability of local authorities to prevent over-crowding, ensure physical distancing standards in public and tourist areas (M = 4.70), and control/regulate access to them (e.g., by using app) (M = 4.27). On the whole, individuals belonging to this group were very interested in travelling to less crowded, less known, and less established tourism destinations (M = 4.50) able to provide them with several outdoor activities and experiences (M = 4.77) and/or with a wide variety of open-air attractions (M = 4.43). Finally, they preferred tourism destinations with a good and easily accessible healthcare system (M = 4.62) and places in which tourism businesses would provide them with face masks and hand sanitiser (M = 4.50).

The “Middle-concerned tourists” (N = 1,120) group was made up of individuals seeking tourism destinations that guaranteed high standards in terms of the sanitisation of public areas (M = 4.43) and healthcare infrastructures and their related accessibility (M = 4.33). The effectiveness by which local authorities could manage social gatherings in order to prevent overcrowding was also considered an important issue by this group (M = 4.17). “Middle concerned tourists” appeared to be relatively neutral when it came to the need to visit tourism destinations that regulate access to public areas in order to properly manage social gatherings and preventing overcrowding (M = 3.56) and/or in places where tourism businesses offer contactless payment systems to contribute towards safety and hygiene standards (M = 3.10). Similarly, they also appeared to be less interested in visiting less known and less crowded places (M = 3.13) and/or places that are rich in terms of open-air tourist attractions (M = 3.19).

Finally, the “Outdoor-driven tourists” (N = 912) group included Italians relatively interested in visiting less known/crowded places (M = 3.77) and tourism destinations that are rich in terms of the outdoor activities (M = 4.60) and open-air attractions (M = 4.22) they offer. However, these individuals appeared to not be particularly interested in the cleanliness and sanitation standards of public and tourist areas (M = 3.41), whether tourism businesses would provide them with PPE (M = 2.78), and whether tourism destinations would make use of appropriate measures and tools to regulate access to tourist areas (M = 2.50) to properly manage social gatherings and avoid overcrowding (M = 2.75).

Furthermore, a series of chi-square tests (χ^2^) were conducted and significant differences were reported to exist among clusters based on gender (X^2^ = 57.882; p < 0.001), age (X^2^ = 49.572; *p* < 0.001), education level (X^2^ = 39.051; *p* < 0.001), occupation (X^2^ = 60.670; *p* < 0.001), type of accommodation (X^2^ = 88.551; *p* < 0.001), and length of stay (X^2^ = 32.696; *p* < 0.001) (Table 5).

**Table 5:** Chi-squared tests

	Chi-squared	*p* value
Gender	57.822	.000
Age	49.572	.000
Level of education	39.051	.000
Employment status	60.670	.000
Type of accommodation you would choose over the next 12 months (2020–2021)	88.551	.000

“Outdoor-driven tourists” is the most gender-balanced cluster (males = 43.1%; females = 56.9%), while “All-round concerned tourists” includes the highest percentage of females (69.4%) when compared to the other clusters. “Middle concerned tourists” is made up mostly by females (59.4%) and has the highest percentage of youngsters (18–24 years old: 12%), whereas “Outdoor-driven tourists” were mostly aged 35–44 (32.2%) or 25–34 years (25%); “All-round -19 concerned tourists” was the oldest cluster, with 43.6% of individuals aged 45 or more (45–54 = 24.9%; 55–64 = 12.9%; over 64 = 5.8%). Finally, “Outdoor driven tourists” had the highest percentage of respondents who were freelance (30.4%) or had a postgraduate or PhD degree (19.2%).

For accommodation type, “COVID-19 concerned tourists” and “Outdoor-driven tourists” appeared to prefer B&B (23.8%) or holiday home accommodation (25.4%), while middle concerned tourists were looking for 4-star (24.1%) or 3-star (16.1%) hotel accommodation. Finally, “Outdoor-driven tourists” had the highest percentage of respondents who preferred long stays (11 nights or more: 20.9%) in comparison to the other clusters, who preferred 4–6 night stays (“All-round concerned tourists”: 41.0%; “Middle concerned tourists”: 39.5%).

Finally, a series of F-tests showed that significant differences existed among clusters based on the geographical scale of travelling, travel party companions, and the use of a certain means of transport (Table 6). Overall, respondents preferred regional (M = 3.94) or domestic tourism (M = 3.76). Furthermore, they reported that they preferred travelling with their family (M = 3.95) and using their own cars (M = 3.85).

**Table 6:** F-Tests

	All-round concerned tourists	Middle concerned tourists	Outdoor-driven tourists	Total sample	F	Sig
	N = 2,507	N = 1,120	N = 912			
I will travel in my region	4.11	3.68	3.78	3.94	48.856	**.000**
I will travel outside my region but in my country/nation	3.74	3.73	3.88	3.76	*4.309*	*.014*
I will visit a foreign country	2.09	2.36	2.88	2.31	105.047	**.000**
I will travel with an organized group (Tour operators or Travel Agencies)	1.63	1.64	1.59	1.62	.728	.483
I will travel with a small group of friends	2.68	2.54	2.72	2.65	4.936	**.007**
I will travel alone	2.15	1.97	2.31	2.14	12.990	**.000**
I will travel with my family	4.05	3.82	3.81	3.95	16.610	**.000**
I will travel by car	4.03	3.66	3.57	3.85	55.966	**.000**
I will travel by train/bus	1.85	1.82	1.99	1.87	6.443	**.002**
I will travel by plane	2.78	2.89	3.07	2.86	12.981	**.000**
I will travel by boat (ferry, cruise ships, etc.)	2.15	2.14	2.21	2.157	.994	.370

Bold character identifies when the p-value is significant

Further analysis of the main differences between clusters revealed that “All-round concerned tourists” were the most interested in experiencing holidays within their region of residency (M = 4.11), with their family (M = 4.05) and using their own car (M = 4.03), when compared to their counterparts. This could be attributed to the fact that individuals within this cluster were the most concerned about COVID-19 health risks. “Outdoor driven tourists” appeared to be the cluster with the highest preference for domestic tourism (M = 3.88) and for travelling by plane (M = 3.073).

### Qualitative findings

Consistent with the PMT and the coping methods identified by Rippetoe and Rogers (1987), two coping modes emerged (Table 7). The first is the adaptive coping mode, which is related to rational problem solving and refers to situations in which respondents were actively willing to adapt their behaviour as a result of the pandemic. In this research, adaptive coping specifically refers to a number of intentions related to changing behaviours with regards to different aspects of travel; from the choice as to whether to travel, to the choice of destination, transport mode, and accommodation, to the behaviours adopted while on holiday and the services used while at a destination. The second coping mode (maladaptive coping) refers to avoidance behaviours but, contrary to the original research applying PMT to health risks (Ben-ahron et al., 1995; Norman et al., 2005; Rippetoe & Rogers, 1987), our study did not find other instances of maladaptive coping modes, as discussed in the literature review. In addition to this, the in-depth analysis of avoidance behaviours allowed us to identify two levels of avoidance: complete (in the case of individuals who did not want to make any change to their behaviour) and partial (for individuals who were only willing to adopt behaviours required by government regulations—i.e., use of PPE, hand sanitisers, and compliance with social distancing rules—but did not want to change any other aspect of their trip).

**Table 7:** Main themes resulting from the qualitative analysis

Coping mode	Themes	# times cited*
Adaptive coping	*Accommodation:*	
	Cleanliness and hygiene	74
	Hotels	13
	Non-hotel accommodation	42
	Private home	12
	Avoid services	20
	Less travel	47
	No holidays	46
	Out of peak season	14
	*Personal behaviour:*	
	PPE and hand sanitising	123
	Social distancing	88
	Booking	19
	Sanitary conditions at destination	34
	*Transport:*	
	Private transport	138
	Avoid public transport	67
	Continue using public transport	37
	*Type of destination:*	
	Avoid going abroad	76
	Continue going abroad	7
	Travel in Italy (near home)	120
	Travel in Italy (nationwide)	121
	Destinations not crowded	158
	Possibility to stay outdoors	68
	Wait until the pandemic is over	39
Maladaptive coping	Complete avoidance	157
	Partial avoidance	41

*The number of times a theme was cited does not refer to the number of people who mentioned a theme because, in many cases, respondents mentioned more than one theme within the same answer

#### Adaptive coping

Among the respondents who decided not to go on holiday or to travel less in comparison to previous years, many mentioned financial issues due to the loss of their job or a decrease in the availability of disposable income, while others mentioned the lack of safety they perceived while travelling during a pandemic (mainly in relation to the fear of contracting COVID-19 while on holiday). This latter point was also raised by respondents who stated that they would have preferred to wait before travelling; for some of them, it was a matter of waiting until the vaccine was available to the population while, for others, it was a case of waiting for the overall situation to improve.

Among those who mentioned that they would have travelled, on a few instances (34), respondents mentioned that they would have chosen a destination if it maintained good sanitary conditions in terms of COVID-19 and had an effective and easily accessible local health system. This was probably due to the fact that, in many cases (241 times), respondents mentioned that they would have chosen a destination in Italy—either close to their city of origin, or nationwide. This was also reflected by those respondents who clearly stated that they would have avoided travelling abroad, not only due to the border being closed at the time of the survey, but also due to the uncertainty of the health support they would have received if they had caught COVID-19 while on holiday.

Apart from being quite specific in terms of their choices of the geographical destination, respondents expressed their willingness to travel to less crowded destinations several times (158 times), as well as places in which it was also possible to practice outdoor activities (68 times). This was related to the fact that, in many cases, respondents felt that, by so doing, they were able to maintain social distancing and maintain a certain degree of safety while on holiday. Hence, this result is similar to recent research that shows a preference in tourists with regards to travelling to less crowded destinations (Aiello et al., 2020). It also confirms results of the quantitative strand of this study.

Not surprisingly, respondents mentioned their willingness to use private transport (i.e., car, motorbike, camper) to go on holiday several times, thus suggesting that this means of transport was deemed more secure than public transport. However, among the answers of respondents who were likely to travel by public transport, air travel was mentioned 11 times and train travel only 8 times; despite the fact that these two transport modes were referred to by respondents as those that were more likely to ensure social distancing while onboard.

As far as accommodation was concerned, respondents frequently expressed their willingness to book accommodation based on the hygiene and sanitation standards of the establishment (74 times).When considering the type of accommodation that respondents would have chosen, non-hotel accommodation (such as B&B and holiday homes) were mentioned several times (42 times), with this preference seen as justified by the fact that non-hotel facilities ensure the respect of social distancing due to the lack of shared areas, as in the case of hotels.

Finally, another theme that respondents cited on several occasions referred to their own personal behaviour while on holiday. Not surprisingly, the use of PPE (e.g., face masks and hand sanitiser) was mentioned (123 times). In this regard, several respondents said that they would have included these objects within their own luggage. In addition to this, behaviours related to maintaining social distancing (88 times) were also mentioned. This is not surprising considering that maintaining social distance has been highlighted as one of the main behaviours to adopt to prevent the COVID-19 infection from spreading in institutional communication across different media. For instance, one respondent stated:

*I think that we have to learn to live with it [COVID-19], so I will pay more attention to social distancing, and face masks will become an integral part of my outfit (Respondent 226)*

Overall, the aforementioned intentions can be easily related to the application of problem solving based on the available information on COVID-19. For this reason those intentions are a representation of adaptive coping, as mentioned in scholarly literature on PMT (Rippetoe & Rogers, 1987).

#### Maladaptive coping

As highlighted in the literature review, according to PMT, maladaptive coping can take different forms, such as avoidance, wishful thinking, fatalism, hopelessness, and religious faith (Ben-ahron et al., 1995; Norman et al., 2005; Rippetoe & Rogers, 1987). However, contrary to previous literature on PMT, in this research, we were only able to identify avoidance as an example of maladaptive coping. Other coping modes were not present in the answers of our sample of respondents. Furthermore, when compared and contrasted with existing literature on PMT, our study identified two degrees of avoidance, namely “complete” and “partial” avoidance.

Complete avoidance refers to a respondent’s intention to not make any change to their future holidays. It must also be pointed out that several respondents declared that they would not have altered their travel behaviour. Some did not specify any reason for this choice, whereas others did, referring mostly to the fact that they would not have changed their holiday habits because, for them, travelling to less crowded destinations with a limited number of people (mainly family and 2–3 friends) and favouring outdoor activities was what they were already doing before the pandemic. Few answers within this theme were from respondents who stated that they did not believe that the situation was as serious as depictions in the media that were believed to exaggerate the reality. One example of complete avoidance is given by the following quote:

*I have always travelled by plane and I will keep doing it. I have always travelled with my family and I will keep doing it. I go to holiday houses and I will keep doing it. […] I am not scared (Respondent 1,409)*

On the contrary, partial avoidance refers to respondents who stated that, although they would not change their way of travelling, they would only adapt to the basic rules necessary to limit the contagion, such as the use of PPE or hand sanitiser and compliance to social distancing rules. For instance, one respondent stated:

*I don’t think that my way of travelling will change, probably I will pay more attention to details, thus I will avoid as much as I can any contacts with other people and I will sanitise my hands (Respondent 250)*

Furthermore, recent tourism literature has shown that holiday spirit is an important element for consideration when applying PMT to the tourism context in particular (Wang et al., 2019). However, in this study, this theme emerged only 34 times. One respondent, for example, stated:

*If the situation is not going to improve as much as I can live my holiday in a serene and free way, then I do not think I will go on holidays. Being constrained by face masks, not having the freedom to visit the places I wish and without limitations would take out from a holiday what it should be about, and I would live it with anguish (Respondent 794)*

## Discussion of merged results

As suggested by existing scholarly literature devoted to mixed methods research, when adopting a convergent parallel mixed method, it is important to merge the two strands of research (Creswell and Plano Clark, 2017) in order to compare, validate, and contrast the related results. (Table 8).

**Table 8:**
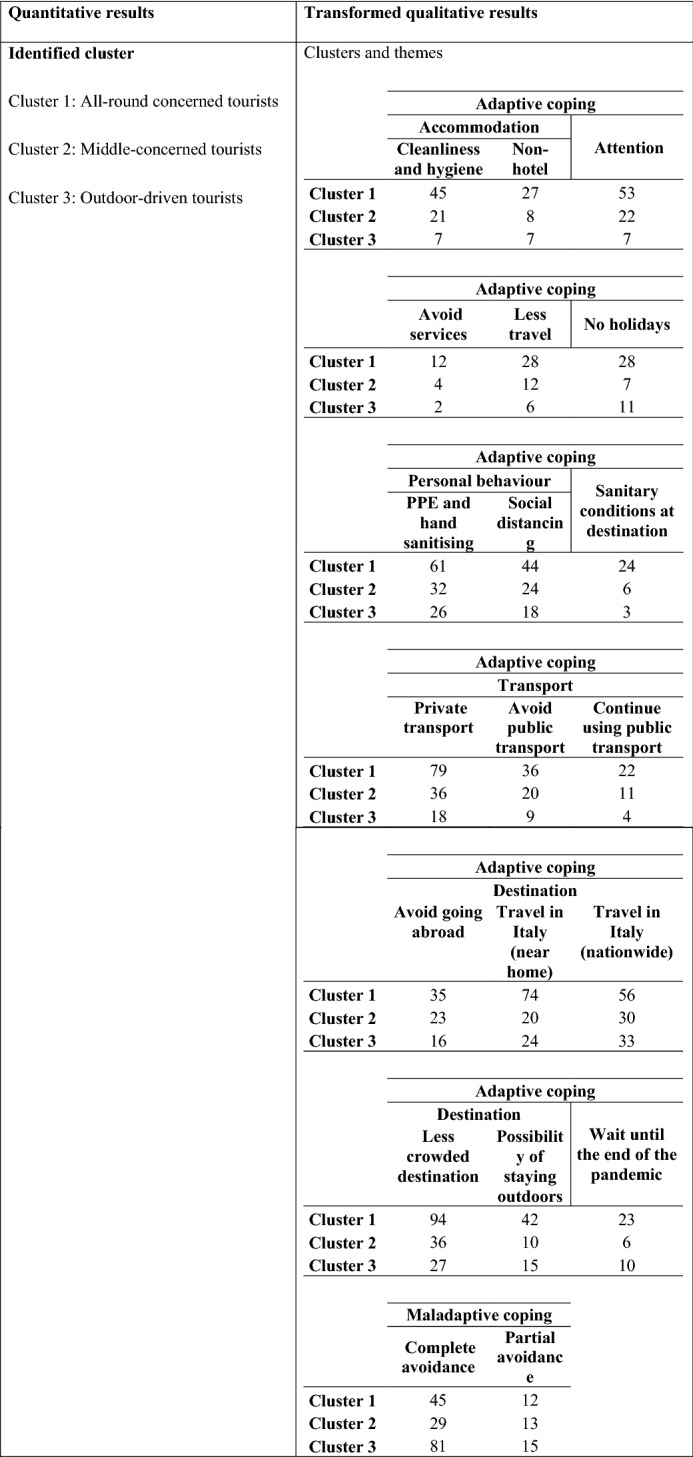
Merged results

“All-round concerned tourists” seems to be made up of individuals who are concerned about the risks generated by the pandemic and, because of this, tend to avoid risky situations (53 times) and would choose destinations that are not crowded (94 times) and provide them with a wide variety of outdoor activities and experiences (42 times). These last two aspects also emerged from the quantitative data, meaning that, to a certain extent, the qualitative results support the quantitative results. While also confirming the quantitative data, the transformed qualitative data allows us to deepen our knowledge of the travel behaviour of “All-round concerned tourists”, showing that respondents are more likely to travel near home (74 times) or domestically (56 times) and are clearly concerned about travelling abroad (35 times). Furthermore, these individuals prefer travelling using private transport (79 times) rather than public transport. With regards to accommodation, people within this cluster pay attention to the cleanliness and hygiene of establishments before booking (45 times) and they favour non-hotel accommodation, such as B&Bs and holiday houses (27 times).

Moreover, this group are likely to use PPE and hand sanitisers (61 times) and practice social distancing (44 times). Overall, we can conclude that this group of respondents tends to adopt adaptive coping strategies, which may be indicative of response efficacy and self-efficacy. The perceived costs associated with the adoption of these adaptive coping strategies are low (Pechmann et al., 2003; Rippetoe & Rogers, 1987). Interestingly, these respondents may also adopt maladaptive coping strategies (45 times). In this regard, it should be noted that the data also shows that some of the people within this cluster tended to travel to less crowded destinations in which they might have practiced outdoor activities; hence, their complete avoidance might be related to the type of travel these people are used to experiencing. In other words, they might have reported no changes to their holiday habits due to the fact that travelling to less crowded places was already one of their travel habits, even before the pandemic, hence their perception of risks due to travelling was low and they believed themselves to be less vulnerable compared to tourists travelling to more crowded destinations (Aiello et al., 2020).

Conversely, “Outdoor-driven tourists” appear to be the least concerned about COVID-19. In this cluster, complete avoidance was shown to occur more frequently in comparison to previous clusters (81 times). Specifically, individuals belonging to this group frequently declared that they would not alter their travel habits, thus showing high levels of confidence. However, also “Outdoor-driven tourists” prefer to travel domestically (57 times) rather than abroad (16 times), as also found for “COVID-19 concerned tourists”. This suggests that travel preferences, in terms of geographical scale, appear to be quite similar regardless of the specific cluster under consideration. Quite interestingly, these respondents mentioned the cleanliness and hygiene of accommodation establishments less frequently when compared to their counterparts. This could be attributed to the fact that “outdoor-driven tourists” may prefer alterative accommodation (i.e., campers, camping, second-homes, etc.), as suggested by existing studies related to ecotourist/outdoor tourists’ behaviours and related accommodation preferences (e.g., Margaryan & Fredman, 2017). Based on PMT, we can suggest that these tourists have a low perception of risk and vulnerability and the rewards that they get from travelling (e.g., being able to travel freely without being subject to too many restrictions) make this group of people more willing to not make any adaptations to their travel, thus resulting in the adoption of maladaptive coping strategies (Floyd et al., 2000; Norman et al., 2005).

## Conclusion

This study sought to contribute to ongoing debates related to travel behaviour in the time of the COVID-19 (or similar) pandemic. Relying on the theoretical lenses of PMT, which have rarely been adopted in marketing (examples of this application include: Pechmann et al., 2003; Tanner et al., 1991) and tourism-related literature (e.g., Wang et al., 2019; Zheng et al., 2021a), a convergent parallel mixed method with a data validation variant was applied to a sample of Italian tourists.

Based on factor analysis, two main factors driving destination choice were identified; namely “PPE, sanitation, & physical distancing” and “Outdoor and under-crowed tourism attractions & destinations”. A cluster analysis identified three clusters (i.e., “All-round concerned tourists”, “Middle-concerned tourists”; and “Outdoor-driven tourists”), which significantly differed from each other in term of socio-demographics (i.e., age, gender, level of education, and employment status) and travel-related variables (preferred accommodation facility and means of transport, geographical scale of travelling, and travel companions). The qualitative research strand further validates these findings while allowing us to further deepen our knowledge on how travel preferences have been altered by the pandemic and how Italians are coping with the related risks. Thus, not only does the qualitative strand of the research corroborate the quantitative findings, but it also allows us to shed additional light on tourists’ behaviour during the current pandemic. Indeed, to the best of the authors’ knowledge, the convergent parallel mixed method has not yet been applied when examining travel behaviour during the pandemic.

These findings are relevant for both researchers and practitioners. On the one hand, they provide insights into relatively under-developed scientific debates on how travellers have reacted to the current pandemic (and, potentially, to any other type of virus that could replicate a similar emphasis on hygiene and safety as well as on physical distancing between people) and, in doing so, further build upon PMT and its application in this peculiar context. Furthermore, according to consumer behaviour theory (Holbrook, 1999), this study also shows that PMT applies in different ways across subjects (i.e., individuals with different socio-demographics) and contexts (i.e., different travel-related characteristics). When compared to existing studies, (Wang et al., 2019; Zheng et al., 2021a), this research not only empirically proves that the ways in which travel preferences are changing are not homogenous, but it also contributes to identifying a certain number of adaptive coping methods that may guide tourists’ decisions during these uncertain times. Furthermore, in this study, two levels of maladaptive coping (complete and partial) have been identified. In previous studies, such a theoretical categorization has neither been proposed nor empirically tested. At the same time, our findings provide further supporting evidence for prior studies (Wen et al., 2020; Zheng et al., 2021a), showing that the more concerned tourists (i.e., “All-round concerned tourists”) tend to adopt more adaptive behaviours than less concerned ones (i.e., “Outdoor-driven tourists”). In doing so, they tend to prefer proximity tourism and use private transport. This seems to confirm that people, when exposed to a disease threat, tend to prefer domestic over foreign destinations both as a way of supporting the domestic economy and related community wellbeing (i.e., the so-called tourism ethnocentrism), avoiding the unknown as much as possible (i.e., the so-called tourism xenophobia). Furthermore, existing literature has shown that perceptions of risks toward diseases may differ between tourists with different sociodemographic characteristics (age, gender, etc.) (Rittichainuwat & Chakraborty, 2009) and our findings further corroborate this idea. Indeed, this research has highlighted that elderly tourists perceive higher levels of risk and have less inclination to travel in comparison to youngsters (Peluso & Pichierri, 2020), while also confirming that women have a higher perception of risks in comparison to men (Neuburger & Egger, 2020). This latter point could be attributed to the fact that women, especially in western societies, are often responsible for decision-making on behalf of their families and children (Khoo-Lattimore et al., 2018; Rojas-de-Gracia, Alarcon-Urbistondo and Casado-Molina, 2019). In addition to this, our research also highlights the usefulness of relying on mixed methods designs in order to deepen and enrich our understanding of complex and dynamic topics—such as the one investigated in this study—in a way that no other studies can when relying on only one singular research strand (i.e., quantitative or qualitative).

This study provides useful information for policy makers, destination managers, hospitality marketers and transport managers attempting to better cope with the current pandemic and effectively manage their service design and related marketing operations. For example, our findings suggest that tourism destinations and related tourism businesses should effectively deliver messages highlighting the COVID-19 procedures implemented to guarantee physical distancing (especially in public and tourist areas), sanitation, and cleanliness, while also promoting the quality and accessibility of the healthcare infrastructure within the area. Such recommendations are particularly relevant when targeting female and senior travellers, who were within the “All-round concerned tourists” cluster that paid particular attention to hygiene, safety, and cleanliness standards at the destination and accommodation facilities, as well as the effectiveness of any social gathering and distancing rules in public areas (e.g., beaches, tourism attractions, etc.). Furthermore, the fact that most Italians appear to be willing to use their own car to go on holiday and to practice proximity or domestic tourism suggests that destination marketers and tourism stakeholders should refocus their marketing campaigns and their media coverage accordingly. In this regard, our findings also suggest that transport managers, especially those operating in the air travel sector, should invest in their methods of ensuring safety on board and inform passengers accordingly, e.g., by establishing new onboard anti COVID-19 procedures, adopting new air filtering systems, and delivering expert-based messages proving the low COVID-19 infection rate onboard if precautions are taken. Moreover, our findings suggest that, in the current scenario, less known and less crowded tourism destinations offering a wide variety of outdoor activities and experiences (e.g., rural areas, mountain destinations, minor “sun, sand, and sea” destinations characterized by less crowded beaches) are particularly attractive to tourists. In particular, destination marketers in these types of locations could promote and position their areas as relatively peaceful places in which individuals can self-critically reflect on themselves and mentally and psychologically recover from the stress of the COVID-19 pandemic (Zenker & Kock, 2020). This suggests that these characteristics should be strongly accentuated in destinations’ promotional activities delivered through offline and online media. On the other hand, destination marketers working in tourism destinations traditionally perceived as being crowded should plan and implement actions aiming to effectively manage tourist flows to ensure visitors’ safety and well-being (Wall, 2020).

Finally, our findings also imply the need to address maladaptive behaviours, especially those of people who purposefully decide not to change their holiday habits during a pandemic. In this case, it is extremely important that nationwide social marketing campaigns relying on different media are delivered and then reinforced by micro-promotion activities ran by tourism stakeholders.

In spite of its theoretical and managerial contributions, this study is not free from limitations. Firstly, it is highly site-specific (i.e., Italy) and is based on a convenience sample, thus rendering our findings less generalizable. Future studies could collect a stratified and representative random sample and could be carried out in other countries. The latter in particular would make cross-cultural comparisons possible and would enable researchers to ascertain whether or not significant differences exist based on the cultural background of travellers (Wen et al., 2020). Secondly, the scales used in this study were not subject to rigorous scale validation because this was not among this research’s purposes. Future studies might consider achieving this specific aim. Thirdly, the typology of coping strategies traditionally associated with PMT (Rippetoe & Rogers, 1987) was not identified in this study. Indeed, although we were able to identify a set of adaptive coping strategies, for maladaptive coping, we were only able to identify avoidance, but not any others (i.e., religious faith, wishful thinking, fatalism, and hopelessness). Hence, future researchers should study these coping strategies in more detail to better clarify whether coping strategies are really made up of two components. In addition to this, avoidance should also be studied in order to facilitate a better understanding of whether the two categories we identified (i.e., complete and partial avoidance) are specific to our sample or if they exist irrespective of the geographical context of research. Fourthly, because this research focused on one section of PMT (i.e., the coping strategies), future research should apply PMT in its totality. In this way, it will be possible to understand the impact that threat appraisal and coping appraisal have on coping strategies (Pechmann et al., 2003). Fifthly, this study focused on analysing travel preferences and travel intentions and, despite data being anonymously collected online, could also have been somewhat subject to social bias (i.e., the tendency for individuals to give socially desirable responses instead of choosing the responses that actually reflect their true feelings (e.g., Fisher, 1993). Future studies investigating actual behaviours would thus be useful. Finally, this study did not analyse how different types of travellers (e.g., leisure *versus* business travellers, group *versus* independent tourists or other special interest tourists such as religious, LGBT + , etc.) might express different types of strategies and behaviours to cope with the COVID-19 pandemic. According to Sigala (2020), this aspect would merit attention in future studies. Our findings provided evidence to show that people exposed to the COVID-19 disease threat tend to prefer domestic over foreign destinations (tourism ethnocentrism and tourism xenophobia). However, the real question that remains to be answered is to what extent this shift will remain stable over time. In this sense, it would be useful to repeat the study over time (i.e., through longitudinal studies) to investigate this further.

## Appendix 1

Factor loadings from factor analysis

**Table:**

	Factor loadings	
	1	2
The tourism destination has good healthcare infrastructures that can be easily accessed when needed	0.602	0.08
Public areas are properly cleaned and sanitised	0.692	0.089
Local authorities know how to manage social gatherings and guarantee social distancing standards in public and tourist areas	0.813	0.113
The access to public areas (e.g., beaches) can be booked (through an app, etc.) to properly manage social gatherings and prevent overcrowding in these places	0.623	0.159
Tourism businesses working within the area allow their customers to use contactless payment methods	0.437	0.283
Tourism businesses working within the area provide their customers with face masks and hand sanitiser	0.662	0.254
The tourism destination is not a crowded, well-known, and well-established tourist area (e.g., rural areas)	0.286	0.542
The tourism destination provides visitors with the option to enjoy several outdoor activities and experiences	0.09	0.653
The tourism destination has several open-air attractions	0.058	0.545

## References

[CR1] Abdullah M, Dias C, Muley D, Shahin M (2020). Exploring the impacts of COVID-19 on travel behavior and mode preferences. Transportation Research Interdisciplinary Perspectives.

[CR2] Addo PC, Fang J, Bakabbey Kulbo N, Li L (2020). COVID-19: Fear appeal favoring purchase behavior towards personal protective equipment. The Service Industries Journal.

[CR3] Aiello F, Bonanno G, Foglia F (2020). On the choice of accommodation type at the time of Covid-19. Some evidence from the Italian tourism sector. Current Issues in Tourism.

[CR39] Auerbach, C. F., & Silverstein, L. B. (2003). *Qualitative Data: An Introduction to Coding and Analysis*. New York: New York University Press.

[CR4] Ben-ahron V, White D, Phillips K (1995). Encouraging drinking at safe limits on single occasions: The potential contribution of protection motivation theory. Alcohol and Alcoholism.

[CR6] Chua B-L, Al-Ansi A, Lee MJ, Han H (2020). Impact of health risk perception on avoidance of international travel in the wake of a pandemic. Current Issues in Tourism.

[CR7] Creswell, J. W., & Clark, V. L. P. (2017). *Designing and conducting mixed methods research*. Sage publications.

[CR5] Del Chiappa G, Pung JM, Atzeni M (2021). Factors influencing choice of accommodation during Covid-19: A mixed-methods study of Italian consumers. Journal of Quality Assurance in Hospitality and Tourism.

[CR1001] Fisher, R. J. (1993). Social desirability bias and the validity of indirect questioning. *Journal of Consumer Research*, *20*(2), 303–315

[CR8] Floyd DL, Prentice-Dunn S, Rogers RW (2000). A meta-analysis of research on protection motivation theory. Journal of Applied Social Psychology.

[CR9] Gursoy D, Chi CG (2020). Effects of COVID-19 pandemic on hospitality industry: Review of the current situations and a research agenda. Journal of Hospitality Marketing and Management.

[CR10] Gursoy D, Can AS, Williams N, Ekinci Y (2021). Evolving impacts of COVID-19 vaccination intentions on travel intentions. The Service Industries Journal.

[CR11] Hair JF, Black WC, Babin BJ, Anderson RE, Tatham RL (2014). Pearson new international edition. *Multivariate data analysis*.

[CR12] He H, Harris L (2020). The impact of Covid-19 pandemic on corporate social responsibility and marketing philosophy. Journal of Business Research.

[CR13] Holbrook MB (1999). Consumer value. A framework for analysis and research.

[CR14] Khoo-Lattimore C, Del Chiappa G, Yang MJ (2018). A family for the holidays: Delineating the hospitality needs of European parents with young children. Young Consumers.

[CR15] Kock F, Nørfelt A, Josiassen A, Assaf AG, Tsionas MG (2020). Understanding the COVID-19 tourist psyche: The evolutionary tourism paradigm. Annals of Tourism Research.

[CR16] Lacroix L, Milliot E (2020). The butterfly effect of COVID-19: Toward an adapted model of commodity supply. COVID-19 and International Business.

[CR17] Lew AA, Cheer JM, Haywood M, Brouder P, Salazar NB (2020). Visions of travel and tourism after the global COVID-19 transformation of 2020. Tourism Geographies.

[CR18] Margaryan L, Fredman P (2017). Bridging outdoor recreation and nature-based tourism in a commercial context: Insights from the Swedish service providers. Journal of Outdoor Recreation and Tourism.

[CR19] Neuburger L, Egger R (2020). Travel risk perception and travel behaviour during the COVID-19 pandemic 2020: A case study of the DACH region. Current Issues in Tourism.

[CR20] Norman P, Boer H, Seydel ER, Conner M, Norman P (2005). Protection Motivation Theory. Predicting health behaviour: Research and practice with social cognition Models.

[CR40] Nunnally, J. C., & Bernstein, I. H. (1994). The Assessment of Reliability. *Psychometric Theory*, 3, 248–292

[CR21] Pechmann C, Zhao G, Goldberg ME, Reibling ET (2003). What to convey in antismoking advertisements for adolescents: The use of protection motivation theory to identify effective message themes. Journal of Marketing.

[CR22] Peluso AM, Pichierri M (2020). Effects of socio-demographics, sense of control, and uncertainty avoidability on post-COVID-19 vacation intention. Current Issues in Tourism.

[CR23] Perić G, Dramićanin S, Conić M (2021). The impact of Serbian tourists' risk perception on their travel intentions during the COVID-19 pandemic. European Journal of Tourism Research.

[CR24] Rather RA (2021). Demystifying the effects of perceived risk and fear on customer engagement, co-creation and revisit intention during COVID-19: A protection motivation theory approach. Journal of Destination Marketing and Management.

[CR25] Rippetoe PA, Rogers RW (1987). Effects of components of protection-motivation theory on adaptive and maladaptive coping with a health threat. Journal of Personality and Social Psychology.

[CR26] Rittichainuwat BN, Chakraborty G (2009). Perceived travel risks regarding terrorism and disease: The case of Thailand. Tourism Management.

[CR27] Rojas-de-Gracia MM, Alarcón-Urbistondo P, Casado-Molina AM (2019). Is asking only one member of a couple sufficient to determine who influences tourism decisions?. Journal of Destination Marketing and Management.

[CR28] Sánchez-Cañizares SM, Cabeza-Ramírez LJ, Muñoz-Fernández G, Fuentes-García FJ (2020). Impact of the perceived risk from Covid-19 on intention to travel. Current Issues in Tourism.

[CR29] Seraphin H, Dosquet F (2020). Mountain tourism and second home tourism as post COVID-19 lockdown placebo?. Worldwide hospitality and tourism themes.

[CR30] Shin H, Kang J (2020). Reducing perceived health risk to attract hotel customers in the COVID-19 pandemic era: Focused on technology innovation for social distancing and cleanliness. International Journal of Hospitality Management.

[CR31] Sigala M (2020). Tourism and COVID-19: Impacts and implications for advancing and resetting industry and research. Journal of Business Research.

[CR32] Tanner JF, Hunt JB, Eppright DR (1991). The protection motivation model: A normative model of fear appeals. Journal of Marketing.

[CR33] Wall G (2020). From carrying capacity to overtourism: A perspective article. Tourism Review.

[CR34] Wang J, Liu-Lastres B, Ritchie BW, Mills DJ (2019). Travellers’ self-protections against health risks: An application of the full protection motivation theory. Annals of Tourism Research.

[CR35] Wen J, Kozak M, Yang S, Liu F (2020). COVID-19: Potential effects on Chinese citizens’ lifestyle and travel. Tourism Review.

[CR36] Wrenn B, Stevens RE, Loudon DL (2007). Marketing research: Text and cases.

[CR37] Zenker S, Kock F (2020). The coronavirus pandemic–A critical discussion of a tourism research agenda. Tourism Management.

[CR38] Zheng D, Luo Q, Ritchie BW (2021). Afraid to travel after COVID-19? Self-protection, coping and resilience against pandemic ‘travel fear’. Tourism Management.

